# Reversion of tumor hepatocytes to normal hepatocytes during liver tumor regression in an oncogene-expressing transgenic zebrafish model

**DOI:** 10.1242/dmm.039578

**Published:** 2019-10-01

**Authors:** Yan Li, Ira Agrawal, Zhiyuan Gong

**Affiliations:** Department of Biological Sciences, National University of Singapore, Singapore 117543

**Keywords:** Hepatocellular carcinoma, HCC, *xmrk/egfr*, Oncogene addiction, Cre/*loxP*

## Abstract

Tumors are frequently dependent on primary oncogenes to maintain their malignant properties (known as ‘oncogene addiction’). We have previously established several inducible hepatocellular carcinoma (HCC) models in zebrafish by transgenic expression of an oncogene. These tumor models are strongly oncogene addicted, as the induced and histologically proven liver tumors regress after suppression of oncogene expression by removal of a chemical inducer. However, the question of whether the liver tumor cells are eliminated or revert to normal cells remains unanswered. In the present study, we generated a novel Cre/*loxP* transgenic zebrafish line, *Tg(fabp10: loxP-EGFP-stop-loxP-DsRed; TRE: CreERT2)* (abbreviated to *CreER*), in order to trace tumor cell lineage during tumor regression after crossing with the *xmrk* (activated *EGFR* homolog) oncogene transgenic line, *Tg(fabp10: rtTA; TRE: xmrk; krt4: EGFP)*. We found that, during HCC regression, restored normal liver contained both reverted tumor hepatocytes (RFP+) and newly differentiated hepatocytes (GFP+). RNA sequencing (RNA-seq) analyses of the RFP+ and GFP+ hepatocyte populations after tumor regression confirmed the conversion of tumor cells to normal hepatocytes, as most of the genes and pathways that were deregulated in the tumor stages were found to have normal regulation in the tumor-reverted hepatocytes. Thus, our lineage-tracing studies demonstrated the potential for transformed tumor cells to revert to normal cells after suppression of expression of a primary oncogene. This observation may provide a basis for the development of a therapeutic approach targeting addicted oncogenes or oncogenic pathways.

## Abstract (precis)

**Summary:** In an oncogene-addicted liver cancer zebrafish model, transformed cancer hepatocytes could be reverted to normal hepatocytes, as shown by Cre-*loxP*-mediated cell-lineage tracing.

## INTRODUCTION

It has been well recognized that many tumors depend on the persistent activation of a single or a few oncogenes to sustain their malignant phenotype, despite their multiple accumulated genetic and epigenetic abnormalities (Weinstein, 2002). This phenomenon is termed ‘oncogene addiction’ and has been supported by abundant experimental evidence using human cancer cell lines and mouse models of cancers, as well as clinical evidence with drugs targeting specific oncoproteins (Weinstein and Joe, 2008). Oncogene addiction provides a rationale for molecular targeted therapy; therefore, understanding the mechanism of oncogene addiction through inactivation of the addicted oncogene holds clinical promises in cancer treatment (Torti and Trusolino, 2011; Weinstein and Joe, 2006).

Using oncogene-driven tumor models in transgenic mice, it has been shown that inhibition of oncogenes could result in rapid tumor regression in many types of tumors, including leukemia, lymphoma, hepatocellular carcinoma (HCC), lung adenocarcinoma, osteosarcoma and breast adenocarcinoma (Bellovin et al., 2013; Felsher, 2010). However, one important question that remains unanswered concerns the fate of tumor cells during tumor regression following the oncogene inactivation. Although proliferation arrest and apoptosis have been found to be a common mechanism in tumor regression, oncogene inactivation seems to have tissue-specific effects (Bellovin et al., 2013). For example, complete tumor elimination has been reported in MYC-induced osteosarcoma and lymphoma (Felsher and Bishop, 1999; Jain et al., 2002), while conversion of tumor cells to a dormant state has been reported for MYC-induced HCC and breast carcinoma, where these dormant cells can revert back to the tumor state upon oncogene reactivation (Boxer et al., 2004; Shachaf et al., 2004). However, direct evidence is still lacking regarding the fates of tumor cells after tumor regression.

Oncogene addiction has also been observed in our previously established HCC models by inducible expression of an oncogene in transgenic zebrafish, where suppression of the oncogene expression results in complete regression of the tumor (Henderson et al., 2013; Li et al., 2012b; Nguyen et al., 2012; Sun et al., 2015). After a few weeks of tumor regression, the tumor livers are generally reverted to histologically normal livers. However, the fate of tumor cells during the regression remains unclear. Elimination of tumor cells through apoptosis is possible as a high level of apoptosis has been observed during tumor regression (Li et al., 2012b). However, a more important question is whether it is possible for the transformed tumor cells to revert to normal cells. In the present study, we adopted the Cre/*loxP*-based cell-tracing method to determine the exact fate of tumor cells during tumor regression in *xmrk* (activated *EGFR* homolog) transgenic zebrafish. We demonstrated that, during liver tumor regression, some of the tumor hepatocytes have undergone apoptosis, while some tumor hepatocytes were transformed into morphologically and transcriptomically normal hepatocytes.

## RESULTS

### Generation of Cre/*loxP* transgenic zebrafish for tracing tumor cell lineage

**Fig. 1.**
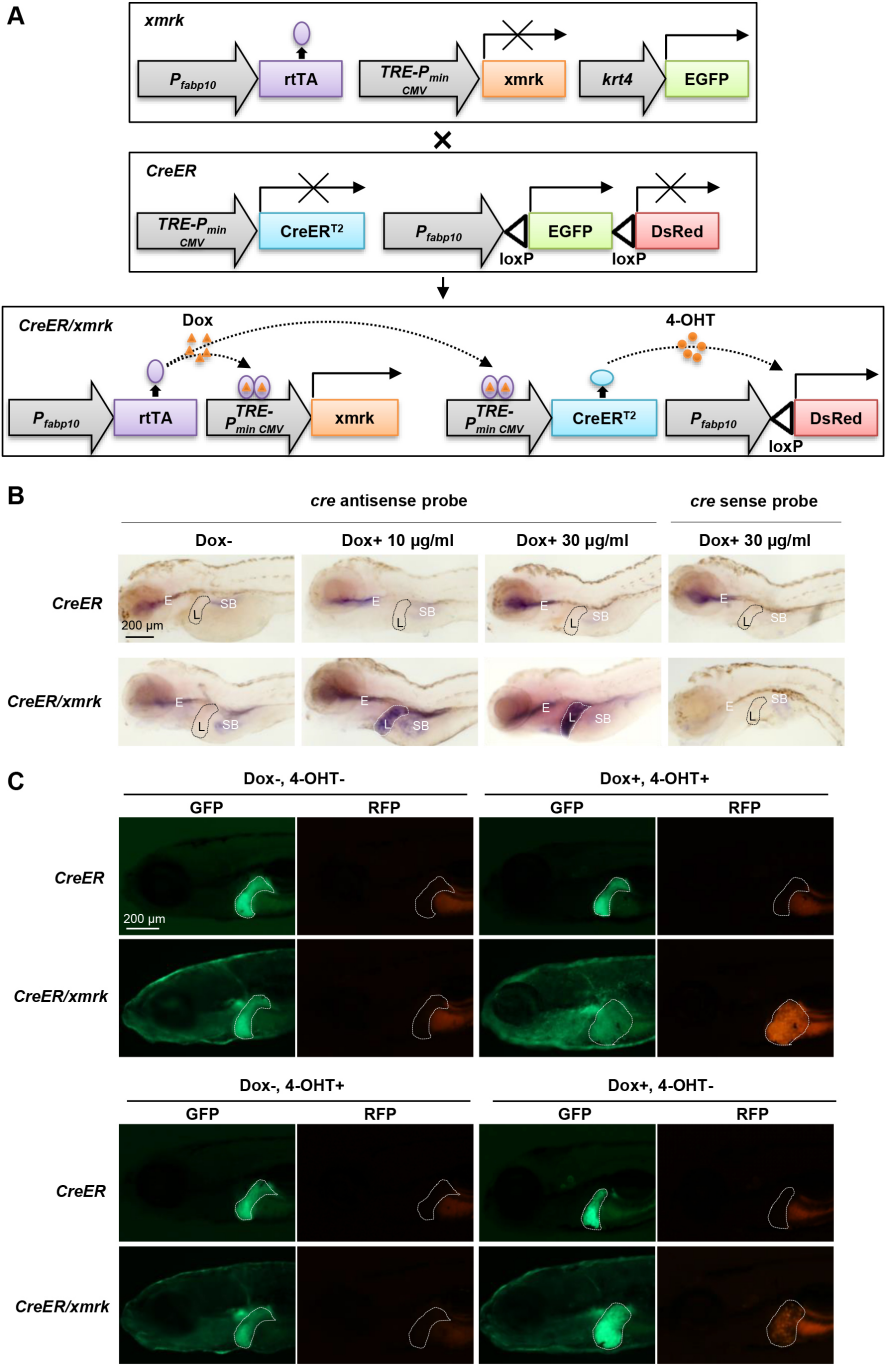
***CreER/xmrk* double-transgenic zebrafish to trace tumor hepatocytes.** (A) Schematics of the transgene constructs for *CreER* and *xmrk* transgenic zebrafish. The *xmrk* construct contains the Tet-on-regulated *xmrk* under the hepatocyte-specific *fabp10* promoter, with the skin promoter *krt4* driving GFP as the selection marker. In the *CreER* construct, the hepatocyte-specific promoter *fabp10* drives the floxed EGFP followed by DsRed. *CreER*^T2^ is controlled by the Tet responsive element (TRE). In the presence of Dox, rtTA will be activated, which in turn activates the *xmrk* oncogene to transform hepatocytes into tumor hepatocytes and also induce the expression of *CreER*^T2^. Subsequent addition of 4-OHT will activate CreER^T2^ to cause *loxP* recombination and thus irreversibly label tumor cells with RFP expression. (B) Liver-specific induction of CreER^T2^ expression. Whole-mount *in situ* hybridization using anti-sense and sense probes against *Cre* mRNA was carried out in *CreER* and *CreER/xmrk* larvae at 4 dpf. No signal was detected with the sense probe in both *CreER* and *CreER/xmrk* larvae. Using the antisense probe, no expression of CreER^T2^ was observed in the liver of *CreER* larvae, either in the absence or presence of Dox (top panels). No expression of CreER^T2^ was observed in the liver of *CreER/xmrk* larvae in the absence of Dox (the far-left bottom panel). Liver-specific induction of CreER^T2^ expression was only observed in the liver of *CreER/xmrk* larvae in the presence of Dox (middle and far-right bottom panels). Livers are outlined in dash lines (L). Non-specific hybridization signals were observed in the regions of swimbladder (SB) and esophagus (E) in some samples. (C) Switch of fluorescent protein expression in livers in *CreER/xmrk* larvae following Dox and 4-OHT treatments at 6 dpf. The livers of *CreER* larvae retained GFP expression in all the four treatment conditions. The livers of *CreER/xmrk* larvae had GFP expression in both Dox– 4-OHT– and Dox– 4-OHT+ treatment conditions, and no leaky expression of RFP was observed (the far-left bottom panel). Leaky CreER activity was observed in the Dox+ 4-OHT– treatment condition. Dox+ 4-OHT+ treatment caused the uniform conversion of the liver for RFP expression in the *CreER/xmrk* fish. Livers are outlined in dash lines.

To trace the fate of tumor hepatocytes, a Cre/*loxP*-based fate-tracing approach was adopted. As shown in Fig. 1A, a novel transgenic line, *Tg(fabp10: loxP-EGFP-stop-loxP-DsRed; TRE: CreER^T2^)* (abbreviated to *CreER*), was established and crossed with the existing *xmrk* transgenic zebrafish to obtain the *CreER*/*xmrk* double-transgenic zebrafish. In these double-transgenic zebrafish, normal hepatocytes would be GFP+ without any chemical induction. Doxycycline (Dox) treatment would activate the transactivator, rtTA, which would in turn activate the *xmrk* oncogene to transform hepatocytes into tumor hepatocytes and also induce the expression of *CreER^T2^*; subsequent treatment of 4-hydroxytamoxifen (4-OHT) would activate CreER^T2^ to cause Cre-mediated *loxP* recombination (Hans et al., 2009) and thus irreversibly label tumor cells with RFP expression. Therefore, temporal control of Cre-mediated *loxP* recombination was achieved by two inducible systems to increase the stringency through the Dox and 4-OHT treatments.

The Dox-inducible and liver-specific expression of *CreER^T2^* was validated by whole-mount *in situ* hybridization. Dox was added to *CreER* and *CreER/xmrk* larval fish from 2 dpf to 4 dpf. Whole-mount *in situ* hybridization was performed using the Cre anti-sense probe at 4 dpf. Expression of *CreER^T2^* was not detectable in the liver of the *CreER* single-transgenic zebrafish either in the absence or presence of Dox (Fig. 1B). In the *CreER/xmrk* double-transgenic zebrafish, expression of *CreER^T2^* was detected only in the presence of Dox and was dose dependent: a high concentration of Dox (30 µg/ml) could induce higher expression of *CreER^T2^* than a low concentration of Dox (10 µg/ml).

The activation of CreER^T2^ by 4-OHT, which would result in *loxP* recombination and color switch of hepatocytes from GFP+ to RFP+, was also validated in larvae (Fig. 1C). Strong and uniform RFP expression was observed in the liver of *CreER/xmrk* fish after Dox and 4-OHT treatments, whereas there was no RFP expression in the *CreER/xmrk* fish without Dox and 4-OHT treatment. The liver of control *CreER* fish remained GFP+ and no RFP could be observed either in the absence or presence of chemical inducers. However, mosaic RFP expression was observed when the *CreER/xmrk* fish were treated with Dox only, suggesting the leakage of CreER activity in the absence of 4-OHT. The leakage of CreER activity in the absence of 4-OHT has been frequently noticed in previous studies (Feng et al., 2017; Herold et al., 2014; Liu et al., 2010). In our study, control of Cre activity was under two inducible systems through the Dox and 4-OHT treatments. Thus, despite the leakage of CreER activity after Dox induction, our system is more stringent than those used in most other studies because, in the absence of both Dox and 4-OHT, no activity of Cre recombinase was detected.

### Transformed HCC cells could be reverted to normal hepatocytes during tumor regression

**Fig. 2.**
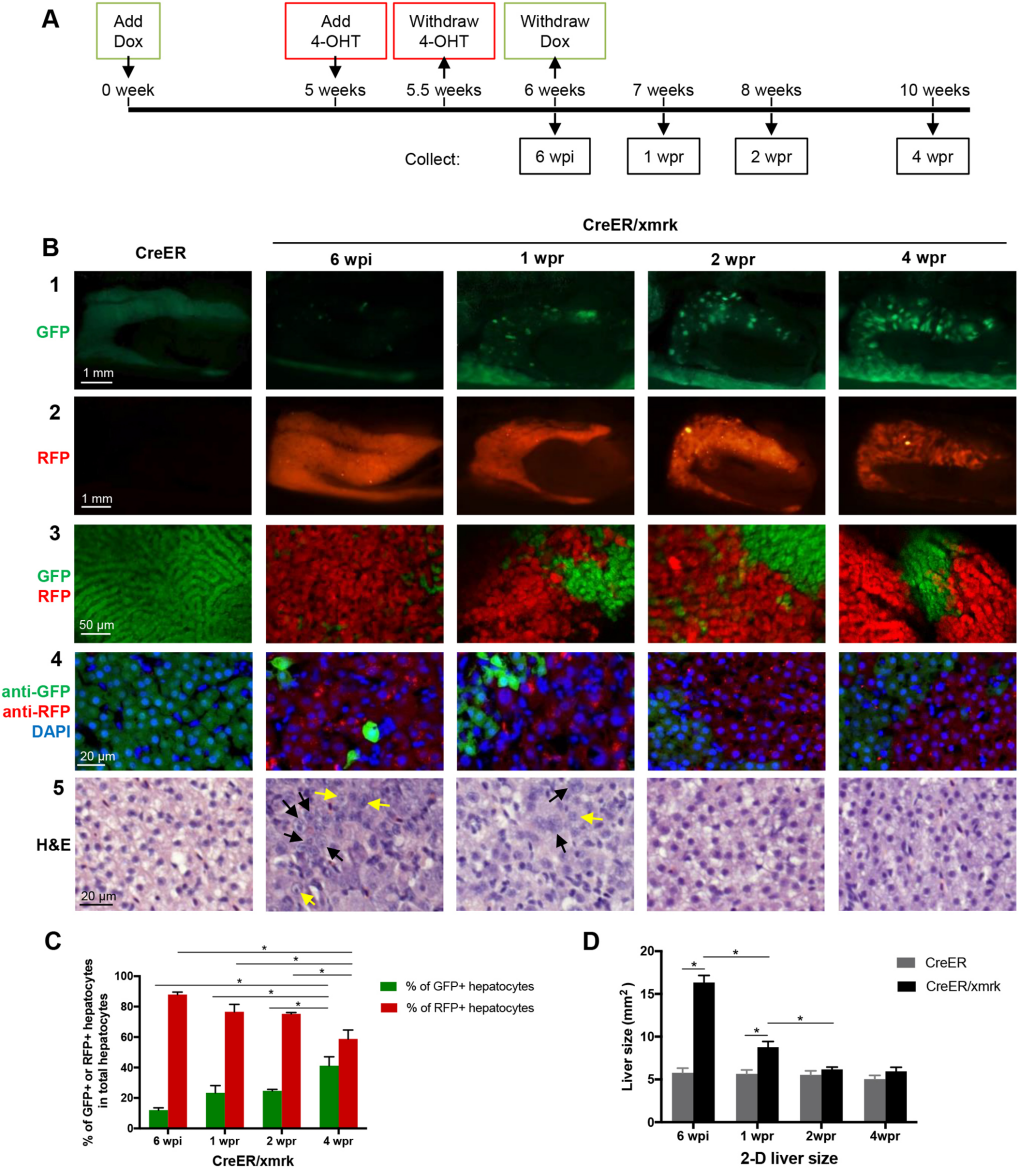
**Tracing liver tumor cells during tumor regression in adult fish.** (A) Flowchart of fish treatment and sample collection. *CreER* and *CreER/xmrk* adult fish (4 mpf) were treated with Dox for 6 weeks to induce HCC. 4-OHT was added at 5 wpi (weeks post-induction) for 3 days to cause *loxP* recombination for marking the tumor cell lineage with RFP expression. Fish were collected at 6 wpi, 1 wpr (weeks post-regression), 2 wpr and 4 wpr for various assays. (B) Fluorescent protein expression and histological analyses of livers. Rows 1 and 2 are representative images of gross observation of GFP and RFP in the livers under a stereomicroscope. Row 3 shows representative confocal images of the fresh liver tissue immersed in phosphate buffer to show the re-establishment of the two-cell hepatic plate structure from tumor-reverted hepatocytes during tumor regression. Row 4 shows representative images of fixed liver sections for GFP expression, RFP expression (stained with anti-RFP antibody) and nuclear staining by DAPI for quantification of cell number. Row 5 shows representative images of H&E staining. Black arrows, large and irregular nuclei; yellow arrows, prominent nucleoli. (C) Quantification of percentages of GFP+ hepatocytes and RFP+ hepatocytes in total hepatocytes based on liver sections from panel B row 3 (*n*=10). (D) Quantification of 2D liver size based on the left lateral view of dissected fish as shown in row 2 of panel B (*n*=10/group). *P<0.05.

Previously, we have demonstrated in *xmrk* transgenic zebrafish that HCC was induced within 3 weeks of Dox treatment and these induced liver tumors were completely regressed and reverted to histologically normal liver phenotype within 4 weeks of Dox withdrawal (Li et al., 2012b). To investigate whether the transformed tumor hepatocytes could revert to normal hepatocytes after tumor regression, the *CreER/xmrk* adult zebrafish [4 months post-fertilization (mpf)] were first treated with Dox for 5 weeks [5 weeks post-induction (wpi)] to induce the expression of *xmrk* and development of HCC (Fig. 2A). 4-OHT was then added at 5 wpi for 3 days to activate Cre-mediated *loxP* recombination and mark tumor cells with RFP expression in the presence of Dox. The fish were rinsed thoroughly with clean water to remove all residual 4-OHT and returned to Dox-alone water for 3 more days to maintain the expression of *xmrk* and the HCC status. Finally, Dox was removed at 6 wpi to suppress *xmrk* expression and initiate tumor regression. Fish were dissected at the HCC stage after Cre/*loxP* labeling, which was at 6 wpi, and at different time points during tumor regression after removal of Dox, including at 1 week post-regression or Dox removal (wpr), 2 wpr and 4 wpr. The *CreER* fish under the same treatment condition were used as the controls.

The expression, cellular localization and stability of CreER were evaluated through immuofluorescent staining using anti-Cre antibody (Fig. S1). In untreated *CreER/xmrk* fish, no expression of CreER was detected. After Dox and 4-OHT induction at 5.5 wpi, CreER was expressed and localized in the nuclei. After 4-OHT withdrawal at 6 wpi, CreER was localized in the cytoplasm but not in the nuclei. After Dox withdrawal at 1 wpr, CreER was basically undetectable. Thus, the function (nuclear localization) of CreER is dependent on the presence of both 4-OHT and Dox, and there was no detectable CreER activity during the tumor regression stage.

In the *CreER* single-transgenic zebrafish, which did not express CreER and the *xmrk* oncogene, the whole liver remained GFP+ and histologically normal (Fig. 2B, far-left column). In the *CreER/xmrk* double-transgenic zebrafish at 6 wpi, hematoxylin and eosin (H&E) staining (Fig. 2B, bottom row) confirmed well-developed and typical HCC features throughout the livers, such as disrupted tissue architecture, large and irregular nuclei, and prominent nucleoli (Lam et al., 2006; Mudbhary et al., 2014). Progressive tumor regression was observed after removal of Dox at 1-4 wpr, consistent with our previous observation in *xmrk* fish (Li et al., 2012b). By 2 wpr, histological examination showed basically normal appearance in cell morphology and the restoration of a two-cell plate structure. By 4 wpr, all fish showed normal liver histology that was indistinguishable from that of control fish. These tumor-regressed fish had no apparent tumor recurrence even after 9 month of Dox removal (data not shown).

In the *CreER/xmrk* fish at 6 wpi, gross observation suggested that the whole liver was uniformly labeled with RFP (Fig. 2B, second row), because Cre-mediated *loxP* recombination had resulted in the excision of the *GFP* gene and the expression of RFP. By examination of the liver sections, we observed that most of the HCC cells were RFP+; only a few cells still showed GFP expression. These GFP+ cells could be either tumor cells that failed to undergo *loxP* recombination or newly differentiated hepatocytes from precursor cells. To determine the two possibilities, we performed gene expression analysis of fluorescence-activated cell sorting (FACS)-sorted RFP+ and GFP+ cells at 6 wpi (Fig. S2). Both GFP+ and RFP+ cells, as well as a population positive for both RFP and GFP (T-RFP+GFP), expressed similar levels of *Cre* and *xmrk*. Genes involved in cell proliferation and tumor development were upregulated in all three cell populations at comparable levels. Genes that are associated with prominent liver function (detoxification metabolism, lipid and glucose metabolism) were downregulated in all three cell populations also at comparable levels. These results indicated that both the GFP+ and T-RFP+GFP cells were molecularly equivalent to RFP+ cells at 6 wpi; thus, it is likely that the GFP+ cells were tumor hepatocytes that did not undergo *loxP* recombination, while T-RFP+GFP cells were tumor cells that had undergone *loxP* recombination but their trace amount of GFP protein had not been fully degraded.

During tumor regression from 1 wpr to 4 wpr, there was a decrease of RFP+ hepatocytes and an increase of GFP+ hepatocytes, indicating the death of tumor hepatocytes (RFP+) and emergence of newly differentiated hepatocytes (GFP+). Interestingly, those newly differentiated hepatocytes (GFP+) appeared as clusters of cells, unlike the discrete distribution of GFP+ hepatocytes in the tumor stage at 6 wpi (Fig. 2B, rows 3-4). By 4 wpr, when tumor regression was completed, the liver size was reduced to one third based on 2D measurement (Fig. 2D), and the liver appeared to be normal (Fig. 2B, row 5). The tumor cell lineage (RFP+ cells) constituted around 58% of the total hepatocyte population at 4 wpr (Fig. 2C), suggesting that some of the tumor hepatocytes were reverted to normal hepatocytes after suppression of *xmrk* oncogene expression. Further evidence for the reversion of RFP+ tumor hepatocytes to normal hepatocytes is the restoration of regular two-cell hepatic plates, which are a typical feature of hepatocyte organization in the normal liver (Fig. 2B, row 3 for 2 wpr and 4 wpr).

Likely due to incomplete *loxP* recombination, a small percentage (∼12%) of tumor cells remain GFP+ (Fig. 2C), and they may contribute to the GFP+ cells during tumor regression. However, these GFP+ tumor cells should behave similarly to RFP+ tumor hepatocytes and most of them should die during tumor regression. As the liver size was reduced to one third during tumor regression based on 2D measurement (Fig. 2C), it is estimated to reduce to ∼20% in volume (3D). Thus, it is likely that at least 80% of tumor hepatocytes died during the tumor regression. Therefore, the GFP+ regressed tumor cells (due to incomplete *loxP* excision) will only contribute to a very small percentage of the GFP+ cells at 4 wpr, and the majority of GFP+ cells should be the newly differentiated hepatocytes.

**Fig. 3.**
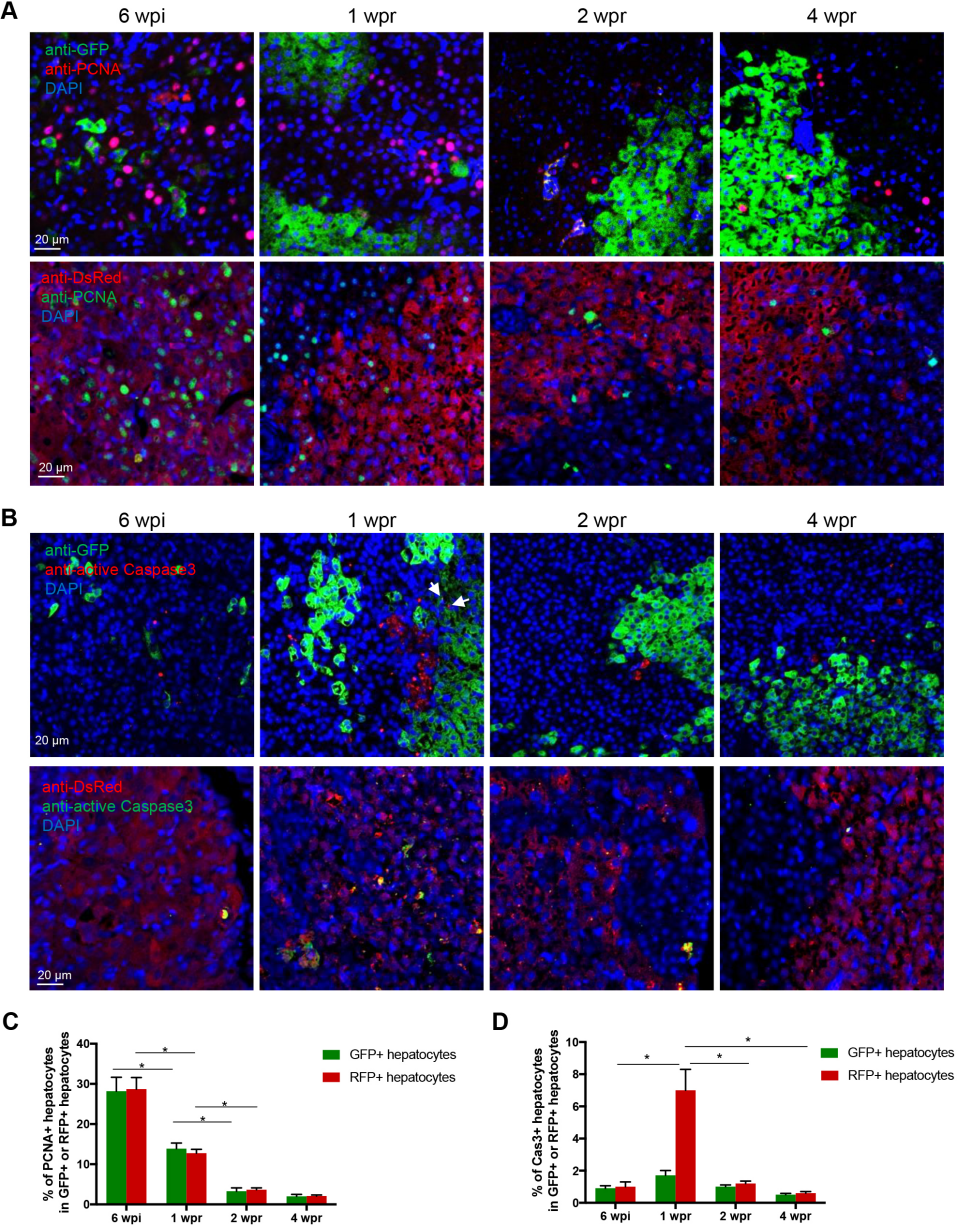
**Proliferation and apoptosis of tumor-reverted hepatocytes and newly differentiated hepatocytes during adult liver tumor regression of *CreER/xmrk* fish.** (A) Representative images of immunofluorescent staining for PCNA in liver sections. RFP and GFP are mainly localized in the cytoplasm, whereas PCNA is located in nuclei. (B) Representative images of immunofluorescent staining for active Caspase 3 in liver sections. Colocalization of cytoplasmic Caspase 3 and RFP/GFP is observed. This experiment is described in Fig. 2. All images are from *CreER/xmrk* fish. GFP and RFP signals were enhanced using anti-GFP and anti-DsRed antibody staining. Arrows indicate apoptotic GFP+ cells at 1 wpr. (C,D) Quantification of cell proliferation (C, PCNA+) and apoptosis (D, active Caspase 3+). Percentages of PCNA+ and active Caspase 3+ cells in the GFP+ hepatocytes and RFP+ hepatocytes are presented. The cells were manually counted from five fields (200-400 cells per field) per fish, ten fish per group. *P<0.05.

To understand the cell population dynamics during tumor regression, proliferation and apoptosis of the tumor cell lineage (RFP+) and newly differentiated hepatocytes (GFP+) were examined by immunostaining against proliferating cell nuclear antigen (PCNA) and active Caspase 3 (Fig. 3A,B). All of the RFP+ cells represented the tumor cell lineage, including the tumor cells and tumor-reverted hepatocytes. However, GFP+ cells should be a mixture of newly differentiated hepatocytes and tumor hepatocytes, due to incomplete *loxP* excision. At 6 wpi, the GFP+ cells were all tumor cells. During tumor regression from 1 wpr, newly differentiated hepatocytes appeared and constituted the majority of the GFP+ cells.

There were equally high portions of RFP+ and GFP+ proliferating tumor hepatocytes (∼28%) at 6 wpi (Fig. 3C). During tumor regression, proliferating RFP+ hepatocytes decreased significantly, indicating the cell reversion process from tumor cells to normal liver cells. Proliferating GFP+ hepatocytes, the majority of which were likely newly differentiated hepatocytes, were higher at 1 wpr than those at 2 wpr and 4 wpr. RFP+ tumor hepatocytes went through massive cell apoptosis during early tumor regression at 1 wpr (Fig. 3D). Both proliferation and apoptosis were returned to basal levels from 2 wpr and were comparable to that in normal liver, indicating a restoration of normal cell cycle in the liver. Thus, the newly differentiated hepatocytes and tumor-reverted cells had equal rates of proliferation and apoptosis after the restoration of normal liver.

### Transcriptomic analysis of tumor hepatocytes, tumor-reverted hepatocytes and newly differentiated hepatocytes

**Fig. 4.**
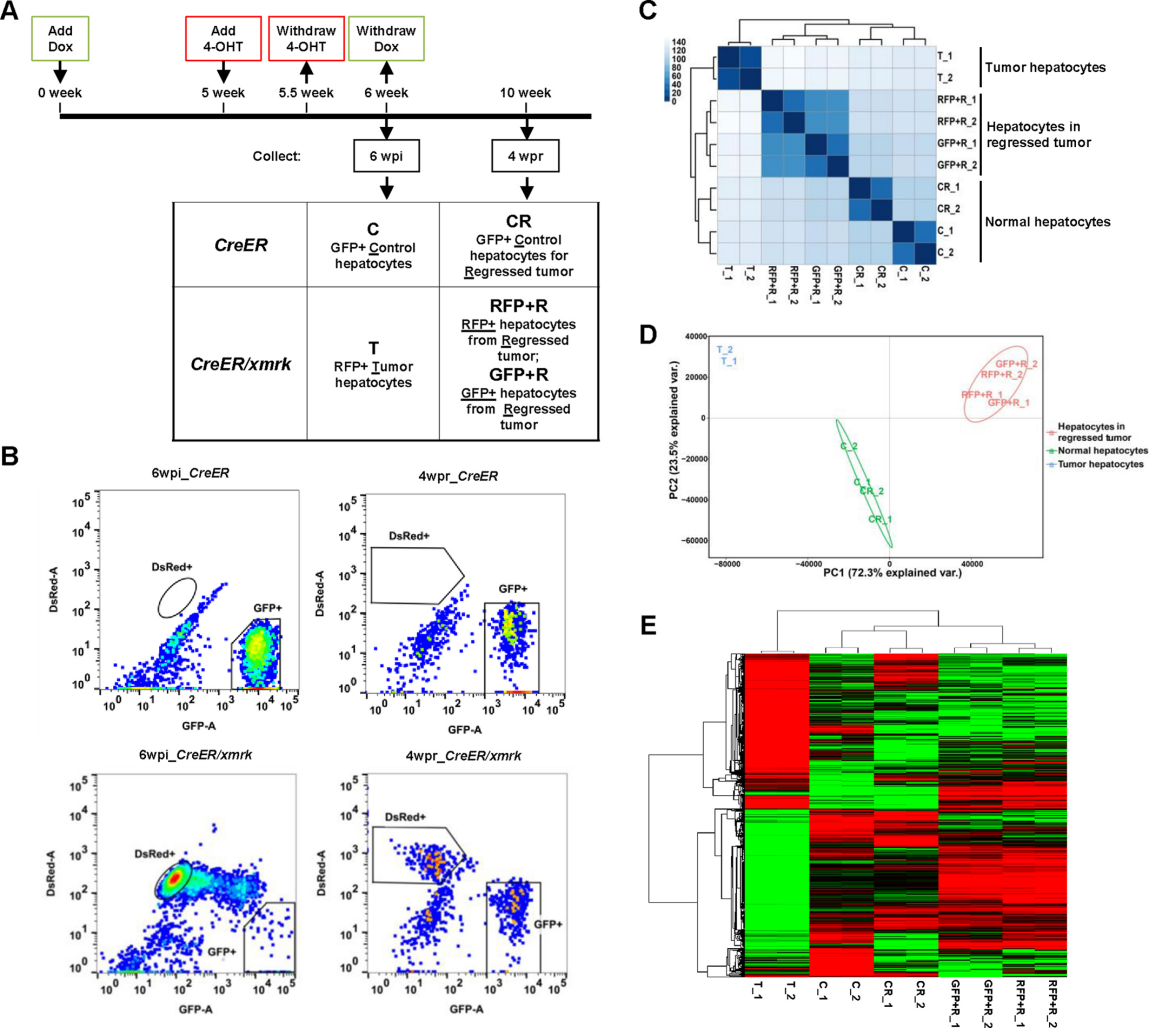
**RNA-seq analyses of tumor-regressed hepatocytes.** (A) Flowchart of fish treatment and sample collection. *CreER* and *CreER/xmrk* adult fish (4 mpf) were treated with Dox for 6 weeks to induce HCC. 4-OHT was added in the last week for 3 days to label tumor cells into RFP+. Livers were dissected at the tumor stage at 6 wpi and at the regression stage at 4 wpr, and subjected to cell sorting for: GFP+ normal hepatocytes from control *CreER* fish at 6 wpi (‘C’) and 4 wpr (‘CR’), RFP+ tumor hepatocytes from *CreER/xmrk* fish at 6 wpi (‘T’), and RFP+ and GFP+ hepatocytes from the regressed tumor of *CreER/xmrk* fish at 4 wpr (‘RFP+R’ and ‘GFP+R’, respectively). (B) FACS profiles of liver cells. Fluorescent-protein-labeled hepatocytes are boxed in each profile. (C) Correlation matrix heatmap showing the Euclidean distance between samples, which was generated using all the expressed genes. Darker color indicates stronger correlation. (D) Principal component analysis (PCA) of gene expression. (E) Hierarchical clustering and heatmap of the differentially expressed genes in various hepatocytes. Gene expression (FPKM) was log2-transformed and median-centered across genes.

As demonstrated in Fig. 2, histological and morphological examination suggested that the tumor hepatocytes could be reverted to normal hepatocytes. However, it is unknown whether the reverted hepatocytes truly resemble normal hepatocytes in their molecular profiles. To answer this question, tumor hepatocytes from *CreER/xmrk* fish at 6 wpi (designated as T for ‘tumor’), RFP+ hepatocytes and GFP+ hepatocytes from the regressed tumor of *CreER/xmrk* fish at 4 wpr (designated as RFP+R and GFP+R, respectively, for ‘regressed’) were isolated by FACS. The control hepatocytes from *CreER* fish at 6 wpi (designated as C for ‘control’) and the control normal hepatocytes from *CreER* fish matching to regressed tumor at 4 wpr (CR for ‘control regressed’) were use as matched controls (Fig. 4A). FACS profiles of different types of hepatocytes are shown in Fig. 4B. In total, five groups of hepatocytes (C, CR, T, RFP+R and GFP+R) were isolated and each group had two replicates from two independent FACS isolations.

The FACS-isolated cells were subjected to RNA-sequencing (RNA-seq) and transcriptomic analysis. The samples were sequenced using the Hi-Seq platform to a depth of 30- to 50-million reads, and 62-76% of the reads could be uniquely mapped to the zebrafish reference sequence database. A total of 12,768 transcripts with a fragment per kilobase million (FPKM) >1 in at least one of the ten samples were considered as meaningfully expressed transcripts (Kim et al., 2017). The Euclidean distance between samples was calculated based on the log-transformed FPKM values of the 12,768 expressed transcripts and presented in a heatmap with clustering (Fig. 4C). The two biological replicates in each sample group were highly similar to each other. The RFP+ tumor-reverted hepatocytes (RFP+R) and GFP+ newly differentiated hepatocytes (GFP+R) were clustered into one branch and shared high levels of similarity. RFP+R and GFP+R were further clustered together with the hepatocytes from normal liver (C and CR), whereas the tumor hepatocytes (T) were clustered into a separate branch, highlighting the fundamental difference between tumor and normal hepatocytes. Principal component analysis (PCA) was performed to assess the overall similarity between samples (Fig. 4D). A PCA plot showed a very similar trend as the heatmap clustering.

Although the RFP+R and GFP+R hepatocytes were very similar and formed one cluster, they did not cluster with the hepatocytes from normal liver (C and CR), suggesting some difference with the hepatocytes from normal liver. Fig. 4E shows a heatmap based on the 3104 genes that were differentially expressed between the tumor hepatocytes (T) and normal hepatocytes (C) [fold change >2, false discovery rate (FDR)-adjusted *P*-value <0.05]. The majority of these tumor-deregulated genes had reverted trends in hepatocytes after tumor regression (RFP+R and GFP+R).

The expression of liver function genes derived from six liver function categories were further examined, including detoxification and xenobiotic metabolism, blood factors, lipid metabolism, glucose metabolism and amino acid metabolism. As shown in Fig. S3, these genes were significantly downregulated in the tumor hepatocytes and their expression was largely recovered in the tumor-reverted cells. Many of them had comparable levels of expression with those in the newly differentiated hepatocytes. This result suggested the destruction of liver function in tumor hepatocytes and the recovery of liver function in the tumor-reverted cells.

**Fig. 5.**
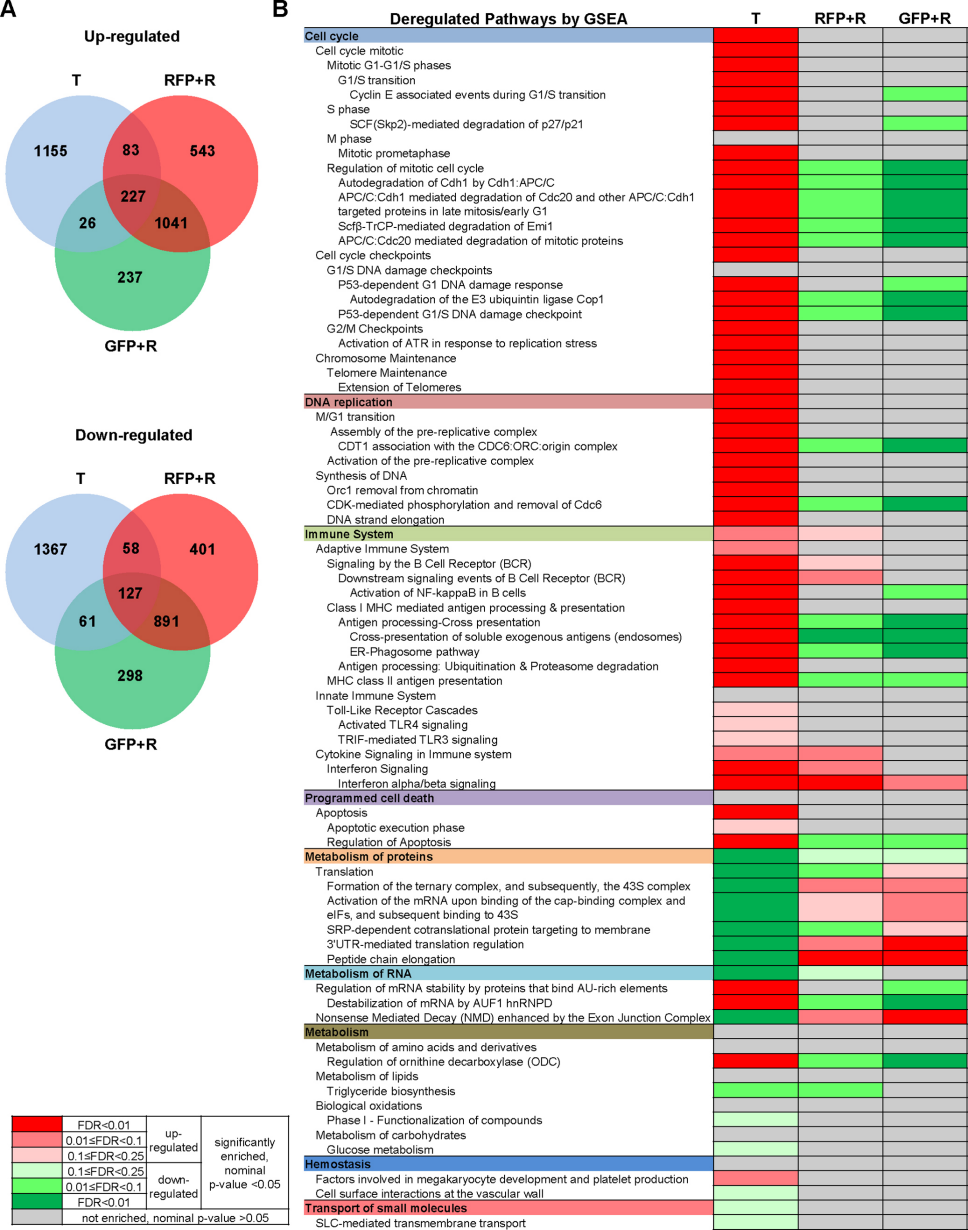
**Deregulated** **genes and pathways in tumor hepatocytes (T), tumor-reverted hepatocytes (RFP+R) and newly differentiated hepatocytes (GFP+R).** (A) Venn diagram showing the overlap between upregulated and downregulated genes in tumor hepatocyte (T), RFP+ tumor-reverted hepatocyte (RFP+R) and GFP+ newly differentiated hepatocyte (GFP+R) samples. (B) Deregulated pathways presented in hierarchical order by GSEA pre-ranked analysis. Red and green indicate up- and downregulation, respectively. The color gradient based on FDR and *P*-value is shown on the left.

The overlapping of the up- and downregulated genes from tumor hepatocytes (T), tumor-reverted hepatocytes (RFP+R) and newly differentiated hepatocytes (GFP+R) (each compared to its matched controls) are presented in two Venn diagrams (Fig. 5A). The tumor-reverted hepatocytes had a much larger portion of overlapping deregulated genes (1268 up- and 1018 downregulated genes) with the newly differentiated hepatocytes than with the tumor cells (310 up- and 185 downregulated genes). The list of differentially expressed genes used for the Venn diagram is presented in Table S2. Selected differentially expressed genes from the Venn diagram were validated using reverse-transcription quantitative PCR (RT-qPCR) analysis. Briefly, four or five genes with different abundance levels (FPKM ranging from 10 to 1000) were chosen from the uniquely up- or downregulated genes in the T, RFP+R and GFP+R groups. As shown in Fig. S4, the RT-qPCR data were generally in good concordance with the RNA-Seq data.

To understand the biological function of the deregulated genes, biological pathways were identified through gene set enrichment analysis (GSEA) using these differentially expressed genes (Fig. 5B). In the tumor hepatocytes, cell-cycle- and DNA-replication-related pathways were upregulated to sustain the high proliferation rate of the tumor. Immune system pathways were also upregulated in tumor hepatocytes, corresponding to the cancer hallmarks of immune activation (Hanahan and Weinberg, 2011). In contrast, the pathways of metabolism of protein, lipids and carbohydrates, and transport of small molecules, were mostly downregulated, also corresponding to the cancer hallmark of reprogramming energy metabolism. Most of these pathways in the tumor-reverted hepatocytes (RFP+R) and the newly differentiated hepatocytes (GFP+R) showed insignificant changes compared to the normal control hepatocytes and a few pathways showed even an opposite trend.

**Fig. 6.**
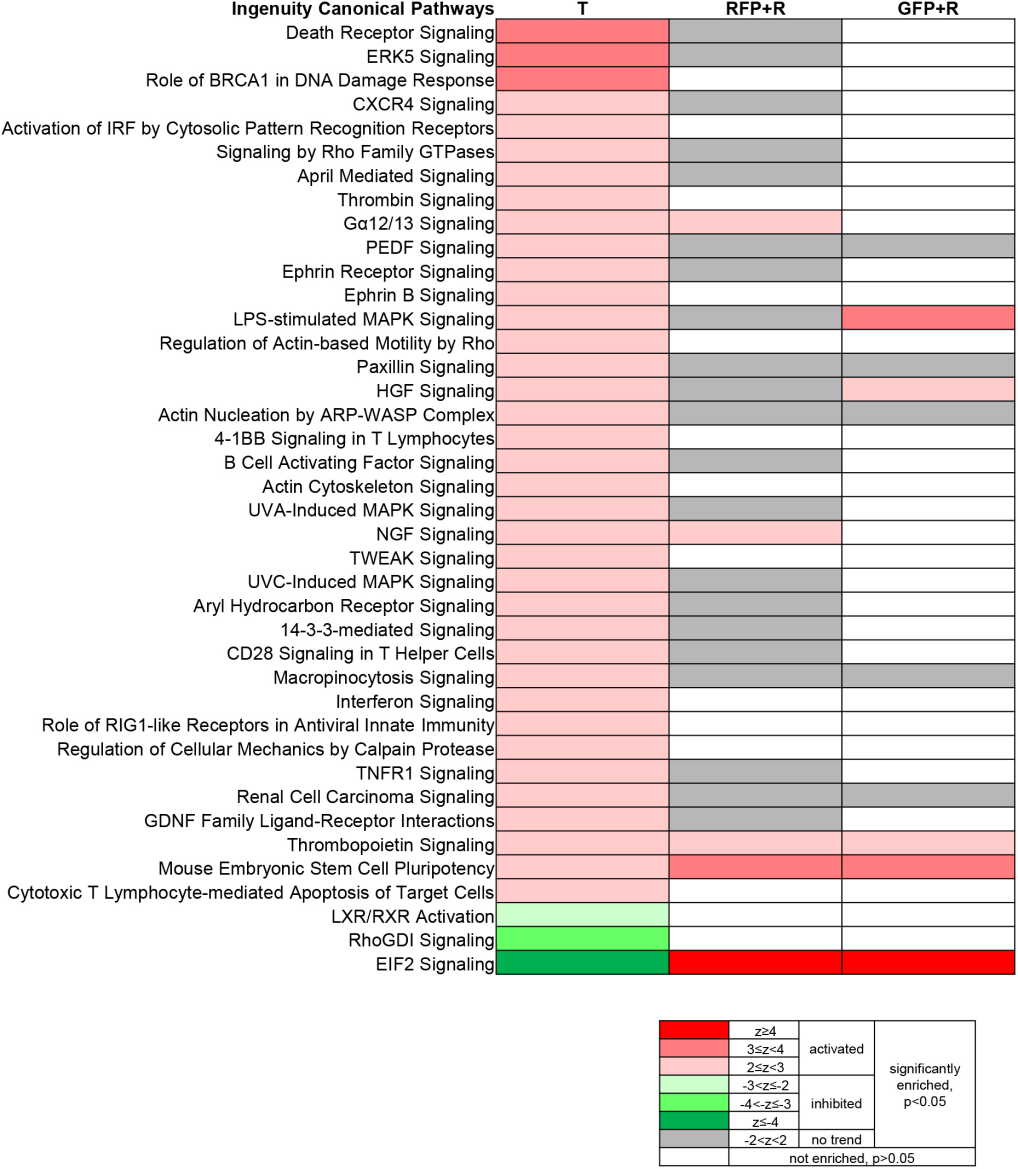
**Deregulated Ingenuity canonical pathways.** Ingenuity canonical pathway analysis was performed using Ingenuity Pathway Analysis (IPA) software. Forty signaling pathways were identified to be enriched and deregulated in the tumor hepatocytes (T). The changes of those tumor-hepatocyte deregulated pathways in reverted hepatocytes (RFP+R) and newly differentiated hepatocytes (GFP+R) are also shown. Red and green indicate activation and inhibition, respectively. Gray indicates enrichment but no predicted trend of activation or inhibition. The color gradient based on *z*-score and *P*-value is shown at the bottom.

To further examine the change of signaling-molecule-centered pathways in the tumor and tumor-regressed liver, Ingenuity canonical pathway analysis was performed using Ingenuity Pathway Analysis (IPA) software (Fig. 6). Forty signaling pathways were identified to be enriched and deregulated (activated or inhibited) in the tumor hepatocytes (T). Although many of these signaling pathways were still predicted to be enriched in the tumor-reverted hepatocytes (RFP+R), the majority of them were no longer predicted to be deregulated (Fig. 6, gray, no trend of activation or inhibition), except for Gα12/13 (α subunits of heterotrimeric G proteins G12 and G13), NGF (Nerve growth factor), Thrombopoietin, mouse embryonic stem cell pluripotency and EIF2 (Eukaryotic initiation factor 2) signaling. Gα12/13 and NGF signaling were activated in the tumor-reverted hepatocytes (RFP+R), but they showed a normal level in the newly differentiated hepatocytes (GFP+R). Lipopolysaccharide-stimulated MAPK and Hepatocyte growth factor (HGF) signaling were activated in the newly differentiated hepatocytes (GFP+R) but not in the tumor-reverted hepatocytes (RFP+R), indicating that these activities may be required in nascent hepatocytes.

## DISCUSSION

It has been robustly demonstrated in many cellular and animal models that tumor development could be induced by a single driver oncogene and could also be regressed when the expression of the oncogene is inhibited: a phenomenon known as oncogene addiction. We also observed robust tumor regression in several transgenic zebrafish models expressing different oncogenes, including *xmrk*, *kras* and *Myc* (Li et al., 2017, 2012b; Nguyen et al., 2012; Sun et al., 2015). The regression could occur even from quite advanced liver tumors with multiple nodules (Li et al., 2017; Sun et al., 2015). Our observations are consistent with data from several oncogene-expressing transgenic mouse models where inactivation of the driver oncogene also causes massive tumor regression (Boxer et al., 2004; Felsher and Bishop, 1999; Jain et al., 2002; Shachaf et al., 2004). Although it has been reported that transformed tumor cells could be reverted to normal cells in *in vitro* cell culture (Kenny and Bissell, 2003; Powers and Pollack, 2016), it remains an enigma whether the oncogene-expressing transformed tumor cells could be reverted to normal cells during tumor regression in an *in vivo* model. Here, we used a genetic recombination system to mark the tumor cell lineage and undoubtedly demonstrated the possibility for tumor hepatocytes to convert to normal hepatocytes both morphologically and molecularly.

However, in clinical human cancers, these cancer cells generally have a long history of development since the initial oncogenic mutations. Generally, clinical human cancer cells have accumulated multiple additional driver mutations to affect multiple molecular pathways to further advance carcinogenesis (Campbell et al., 2017; Gomez et al., 2018). Regression of human clinical cancers may require blocking multiple oncogenes or molecular pathways. In our zebrafish model and other transgenic animal models for tumor regression, it is likely that there are not enough mutations accumulated and the tumors are still addicted to the initial driver oncogene. Therefore, the tumors still rely on the initial driver oncogene for growth and survival, and inhibition of this specific oncogene is sufficient to halt tumorigenesis (Torti and Trusolino, 2011).

The molecular mechanisms for the reversible fate of tumor cells in our zebrafish model remain unknown, but transcriptomic analyses may provide some hints. Tumor signaling pathways that remain activated in the tumor-reverted hepatocytes (RFP+R) but not in the newly differentiated hepatocytes (GFP+R) could be of particular interest. GSEA pathway analysis showed that, when the tumor hepatocytes were reverted to normal hepatocytes during tumor regression, the upregulated cell cycle and DNA replication pathways were generally inhibited while downregulated metabolism pathways were generally reverted. However, immune system pathways showed some interesting changes. On one hand, both class I and II antigen presentation pathways were inhibited, suggesting the decreased production of tumor antigen. On the other hand, the B-cell receptor (BCR) signaling and cytokine signaling in the immune system remained activated in the reverted hepatocytes but not in the newly differentiated hepatocytes. BCR signaling activates multiple signaling cascades involving kinases, GTPases and transcription factors, and contributes to survival and proliferation. While activation of B cells in the tumor microenvironment is known to promote the progression of various cancers, including HCC (Hetta et al., 2016), it is possible that the continuously activated immune pathways contributed to the survival of tumor hepatocytes during tumor regression. Ingenuity canonical pathway analysis revealed that the Gα12/13 and NGF signaling, which were upregulated in tumor hepatocytes, retained their activation state in tumor-reverted hepatocytes but not in the newly differentiated hepatocytes. Gα12/13 signaling is known to promote oncogenesis and tumor cell growth (Juneja and Casey, 2009). NGF has been reported to be involved in tumor growth and invasion, by promoting cell proliferation and survival (Rasi et al., 2007). Therefore, these activated signaling pathways may also contribute to the survival of tumor hepatocytes during tumor regression.

Spontaneous tumor regression is rare in human clinical observations and so far only hundreds of cases have been reported, with an estimated rate of one in every 60,000-100,000 cancer patients (Arora and Madhusudhana, 2011; Chin et al., 2018; Everson, 1964). The feasibility of conversion of tumor cells into normal cells by inhibition of addicted oncogene(s) or pathway(s) provides a basis for development of therapeutic approaches in cancer treatment. One critical factor is identifying the primary addicted oncogene(s) in a cancer, as different approaches are needed to treat cancers caused by different oncogenes. Thus, targeted therapy, as the foundation of precision medicine, could be designed to interfere with the identified driver oncogenes. Development of effective targeted therapy drugs is of particular importance for HCC patients since they are often diagnosed at late stages and have already lost the opportunity of surgical resection (Villanueva and Llovet, 2011). Currently, only two drugs, sorafenib and regorafenib, which target tumor angiogenesis and cell proliferation, have been approved by the US Food and Drug Administration (FDA) for treatment of HCC patients. In addition to its therapeutic benefits to some HCC patients, sorafenib could even cause complete regression of HCC, which has been reported in a few cases (Kim et al., 2017). However, the cytological mechanism by which a complete response was achieved is still unclear, although tumor apoptosis and hypoxia due to vascularization inhibition are known to be involved (Kourtidis et al., 2015). Based on our observation in the zebrafish model, conversion of tumor cells into normal cells could be a possibility during tumor regression in these treated human HCC patients.

Many targeted therapies for HCC are being tested in clinical trials, which target VEGFR, PDGFR, MET, mTOR, CTNNB1 (β-catenin), EGFR, etc. (Ding et al., 2017). The main problem in targeted therapy is the development of resistance to the drug that blocks the target molecule; in particular, resistance to anti-EGFR therapy is frequently observed (Chong and Jänne, 2013). Studies of the resistance mechanisms to EGFR-targeted drugs suggested that concomitant activation of other signaling systems in the cancer cells may be one of the reasons (Berasain and Avila, 2014). Our pathway analyses of the tumor cells (T), in which the *xmrk* oncogene, an activated EGFR homolog (Gómez et al., 2001), was activated, and tumor-reverted cells (RFP+R), in which the oncogene was repressed, suggested some activated signaling in tumor cells, including death receptor signaling, ERK5 signaling, CXCR4 signaling, Ephrin receptor signaling and HGF signaling. These signaling pathways have been reported to be co-activated or have cross-talked with EGFR in HCC and other tumor types (Al Zobair et al., 2013; Berasain and Avila, 2014; Hoang et al., 2017; Zhu et al., 2017), and could consequently promote tumorigenesis. Thus, our observations in this study are consistent with the concept that a therapy to target multiple receptor signaling pathways in tumors could be developed to overcome drug resistance.

Our results suggested that tumor regression was accompanied by the death of tumor hepatocytes, reversion of RFP+ tumor hepatocytes and emergence of GFP+ newly differentiated hepatocytes. We hypothesize that those GFP+ newly differentiated hepatocytes originated from liver progenitor cells because they appeared as clusters of cells. Currently, liver progenitor cells are not well studied in zebrafish and remain controversial in mouse studies. Recently, it has been shown that, after extreme liver damage, liver progenitor cells derived from the biliary epithelial cells could give rise to hepatocytes (Choi et al., 2014; He et al., 2014; Lu et al., 2015; Raven et al., 2017). The origin of new hepatocytes during tumor regression remains an interesting question to be addressed in future with the aid of new lineage-tracing tools, as promotion of the differentiation of new functional hepatocytes during tumor regression and other liver diseases holds therapeutic potential.

In this study, HCC regression was investigated using a zebrafish liver tumor model driven by a fish oncogene, *xmrk*, an activated EGFR homolog (Gómez et al., 2001). Regression of HCC following inhibition of the *xmrk* oncogene could be useful to model the targeted therapy of human HCC dominated by the EGFR pathway. We have shown that, during the tumor regression following suppression of *xmrk* oncogene expression, tumor hepatocytes could be reverted to normal hepatocytes. Thus, our observation should provide a basis for the development of therapeutic approaches by targeting addicted oncogenes and pathways.

## MATERIALS AND METHODS

### Zebrafish maintenance

All zebrafish experiments were carried out in accordance with the Guide for the Care and Use of Laboratory Animals of the National Institutes of Health and the protocol was approved by the Institutional Animal Care and Use Committee (IACUC) of the National University of Singapore. The *xmrk* transgenic zebrafish [*Tg(fabp10:rtTA; TRE2:xmrk; krt4:GFP)*] were previously generated (Li et al., 2012b) and this transgenic line comprises a Tet-On system for inducible and liver-specific expression of oncogenic *xmrk*. The *Cre* transgenic fish [*Tg(fabp10:loxP-GFP-loxP-DsRed; TRE2:CreER^T2^)*] were generated in the present study by injection of the *pDs-fabp10-loxP-EGFP-loxP-DsRed-TRE-CreERT2* plasmid with AC transposase mRNA at the one-cell stage and is referred to as *CreER* in this report.

### Doxycycline (Dox) and 4-hydroxytamoxifen (4-OHT) treatments

To induce *xmrk* and *CreER*^T2^ expression and HCC formation in adult zebrafish, 60 μg/ml Dox (Sigma-Aldrich) was used to treat 4-month-old fish for 6 weeks. Larval fish were treated with 20 μg/ml Dox from 3 days post-fertilization (dpf) to 8 dpf. To induce Cre-mediated *loxP* recombination, 1 μM 4-OHT (Sigma-Aldrich) was added to the adult or larval fish for 3 days before Dox withdrawal.

### Histological and cytological analyses

Fish samples were fixed in formalin solution (Sigma-Aldrich) and sectioned at 5-µm thickness using a microtome, followed by hematoxylin and eosin (H&E) (Sigma-Aldrich) and immunofluorescent staining. Immunofluorescent staining was carried out as previously described (Yan et al., 2015). The primary antibodies used included rabbit anti-PCNA at a dilution of 1:200 (Santa Cruz Biotechnology, SC-7907), rabbit anti-Caspase 3 at a dilution of 1:200 (BD Biosciences, 559565), mouse anti-GFP at a dilution of 1:500 (Millipore, MAB3580), rabbit anti-RFP at a dilution of 1:100 (Abcam, ab62341), mouse anti-DsRed at a dilution of 1:100 (Santa Cruz Biotechnology, sc390909) and mouse anti-Cre antibody at a dilution of 1:200 (Abcam, ab24607).

### Isolation of hepatocytes by FACS

Five adult zebrafish livers were sampled and pooled. The livers were dissociated into single cells through enzymatic digestion with 0.25% trypsin (Sigma-Aldrich) and filtration with a 40-μm mesh (BD Biosciences). The cells were subjected to FACS using a cell sorter (Beckman Coulter) on the basis of GFP or DsRed expression.

### RNA extraction, cDNA amplification, qPCR and whole-mount *in situ* hybridization

Total RNA was extracted using TRIzol reagent (Invitrogen), followed by cDNA synthesis using a Transcriptor First Strand cDNA Synthesis Kit (Roche). The cDNA was used for real-time quantitative PCR (RT-qPCR) with a LightCycler 480 SYBR Green I Master (Roche). Genes of interest were amplified for 40 cycles (95°C, 20 s; 65°C, 15 s; 72°C, 30 s). The sequences of primers used are presented in Tables S1 and S3. Whole-mount *in situ* hybridization was carried out using digoxigenin-labeled sense and antisense RNA probes for the *Cre* gene as described (Li et al., 2012a).

### RNA-seq and bioinformatics analysis

Total RNAs were submitted to Novogen, Hong Kong, for library preparation and HiSeq PE150 sequencing. For library preparation, mRNA was enriched from total RNA by Oligo (dT) beads and rRNA was removed through the Ribo-Zero kit (Illumina). The mRNA was then fragmented randomly, followed by cDNA synthesis using random hexamer primer and second-strand synthesis. After a series of terminal repair, A ligation and sequencing adaptor ligation, the double-stranded cDNA library was completed through size selection and PCR enrichment. Sequence reads were mapped to the zebrafish reference genome with TopHat. Transcript assembly was performed with Cufflinks and Cuffmerge. Differentially expressed genes were determined with Cuffdiff (Trapnell et al., 2012). An FPKM cutoff of 1 in at least one of the ten samples was applied to capture meaningfully expressed transcripts (Kim et al., 2017). Differentially expressed genes were determined by a fold change >2 and FDR-adjusted *P*-value <0.05. Hierarchical clustering was performed using Cluster 3.0 and heatmap was generated using TreeView (Mortazavi et al., 2008). Euclidean distance between samples was calculated and visualized in a heatmap in the R statistical software environment with packages from Bioconductor (Love et al., 2015). PCA was performed using the prcomp function in R and plotted using the ggbiplot package in R with FPKM values. GSEA pre-ranked analysis was performed to identify deregulated pathways using the curated canonical pathways from the MSigDB (Molecular Signature Database) and the differentially expressed genes ranked by log-transformed fold change (base 10) (Subramanian et al., 2005). Ingenuity pathway analysis was carried out for the differentially expressed genes (Ingenuity Systems; www.ingenuity.com).

### Statistical analysis

Results were plotted and statistical analysis was performed using Graphpad Prism software version 7.0. Data were presented as mean±standard error of mean (s.e.m.). Statistical comparisons of two or more groups with two variables were analyzed by two-way ANOVA with Turkey's multiple-comparisons test (all the remaining graphs).

## Supplementary Material

Supplementary information
