# Supplementary material for: Reversion of tumor hepatocytes to normal hepatocytes during liver tumor regression in an oncogene-expressing transgenic zebrafish model
Source: Dis Model Mech. 2019 Oct 17;12(10):dmm039578. doi: 10.1242/dmm.039578 (PMC6826027; doi:10.1242/dmm.039578)
Supplement: Supplementary information [file dmm-12-039578-s1.pdf]

Table S1. List of PCR primers for comparing RFP and GFP cells in Fig. S2

Gene Symbol	Accession ID	Forward Primer	Reverse Primer
cre	YP_006472	GCCAGCTAAACATGCTTCAT	TAACATTCTCCCACCGTCAG
xmrk	X16891	GACTGCTGCAACGAACACTG	CATTCCTTGACACAAGCGGC
ccnd1	NM_131025	TTCCTTGCCAAACTGCCTAT	GGTGAGGTTCTGGGATGAGA
ccng1	NM_199481.1	GCTCAACTGGAAGGTCAAGG	CAGGGCCAGAAGAGACAAAG
cdk1	NM_212564.2	CTCTGGGGACCCCTAACAAT	CGGATGTGTCATTGCTTGTC
cdk2	NM_213406.1	CAGCTCTTCCGGATATTTCG	CCGAGATCCTCTTGTTTGGA
pcna	NM_131404.2	GGCAACATCAAGCTCTCACA	TGCAATTTTGTCCTCAACCA
mcm2	NM_173257.2	GCTACGACCCGTCTCTAACG	CGTCGAATGTGTTGGGAAGC
pold4	NM_001256200.1	GAACCCCCACAACTGAGTGA	TGTACTCTGGGTCAGTGTCCT
tp53	NM_001271820.1	GCTTGGTGCTGAATGGACAAC	CGTTTGGTCCCAGTGGTGG
cyp1a	NM_131879.2	TGCGAAGACCGAAAACTGGA	GTGCGATCCTTCCCGATCTT
adh5	NM_131849.2	AGCTCTTCCACTTCATGGGC	ACTTGCAGCCCATTACGACA
adh8a	NM_001001946.4	AAGCGAGGTTTTCCTGTCGT	GAAGGTGCTGGTTCCCATGA
apoa1b	NM_001100144.2	AGTCTCTTCCCAGACCAGCC	GGTGAGTGAGGGACTTGTGT
apoa2	NM_001130586.1	GAGGCTTGTCAATGCTTGGG	ATTCAGGCGAGCTTTGTCCA
fabp3	NM_152961.3	GGACGGTAAAGAGACGACCC	GAAACGGTGCAAAGGAGAGC
pklr	NM_201289.1	GGACAACCGTGTCACCTTCG	CAGGAGGCTGTCAATCAAAGAC
pdhb	NM_213154.1	TGCTTGGTACGGTCACTGTC	CCATTCGGGAGTGAGACACC
aldob	NM_194367.3	GCCACTGAGAAGGTCCTAGC	CACTCTGACCCCCAGAGAGA
gapdh	NM_001115114.1	GGGCTGCCAAGGCTGTAGGC	GGACACAACCTGGTGCTCCGTG

Table S2.

Click here to Download Table S2

Table S3. List of PCR primers for validation of RNA-seq data in Fig. S4

Type	Gene symbol	Forward Primer	Reverse Primer
unique up_T	*ccng1*	GGTGTGCAGGGCAGAAGTAA	GTCCACAGAGTTTGGGCTGA
unique up_T	*dynll1*	AGTCGCTGTTTTCTCCGTGT	GCCAGGTGGGGTTGTACTTT
unique up_T	*cox6a1*	CGAGTTCGTACCCTACAGCC	ACAAACTGGACAGGCGTCAT
unique up_T	*b2m*	TTGTCTGCTTGGCTCTCTCG	CCTGTCCGTTCTTCAGCAGT
unique up_T	*rbp5*	ATCTGGCGGCTTTGGATGTT	CGTAGTTTGGCACTTTCGCC
unique up_RFP+R	*adh8a*	AAGCGAGGTTTTCCTGTCGT	GAAGGTGCTGGTTCCCATGA
unique up_RFP+R	*fbp1b*	AGCCATTTCCACTGCTGTCA	AGAGCCATCCAGAGGGTCAA
unique up_RFP+R	*C7*	ATGTCCAAGCGGAATGCAGA	CTGGCAGACACTCATTCGGT
unique up_RFP+R	*agt*	GAGGGAGCCAGTCTACAGGA	GCCCTCCGTCATCTCAAACA
unique up_RFP+R	*cetp*	CTGTTCCTGGTTGTCGCTCT	TGAATCTCTAAATTGTGGAATCCGT
unique up_GFP+R	*eef1g*	GCAGTGGGTCAGTTTTGCTG	GACGCAGGTCACAAACCAAC
unique up_GFP+R	*rpl39*	GTCGCACAAGACCTTCAGGA	CTGGCAGGGAACAGATGGAG
unique up_GFP+R	*tm4sf4*	GTCCTCTGCAACATCCTGCT	CGCCGCAAACAGAATAGAGC
unique up_GFP+R	*vdac1*	AGGAACCGCATCACACAGAG	CTGGTATTTGGCTGCGATGC
unique up_GFP+R	*nlk1*	CTGCAAACGTGTGTTCAGGG	GTGGAGAATGCCTGCTGAGT
unique down_T	*rpl19*	CTTTCCGCTGACCACCAGC	TGACGACCCTTCCTTCGTG
unique down_T	*hgd*	CTGATCACACCTTTCGGCCT	GACGCTGACAGGTCTCCAAA
unique down_T	*adh5*	AGCTCTTCCACTTCATGGGC	ACTTGCAGCCCATTACGACA
unique down_T	*cyp1a*	TGCGAAGACCGAAAACTGGA	GTGCGATCCTTCCCGATCTT
unique down_RFP+R	*dap*	AAGACAACGACGCGACTCTT	GACCACAACAGGCAGCTTTG
unique down_RFP+R	*sord*	CTGGTCGAGTGGTGAAGGTC	CTGACAGGGGCTCAATCAGG
unique down_RFP+R	*aco2*	CCTGGGAGTTGAAGTGTCCC	TGGCAATTTCTCCTCGACCC
unique down_RFP+R	*nipal4*	AAAAAGAAGGCACTGCTGCG	GCAGCCCAATTTTCCAAGCA
unique down_RFP+R	*psmd1*	AAAACGGCAGACGCTAAGGA	CCCAGTTTGTTGCTCTTGCC
unique down_GFP+R	*sgk1*	GCTGGGCTTCACTGATGACT	CCATAGCAGGGGCGTAAGAG
unique down_GFP+R	*cyb5b*	CACGGAGTGAAAATGGGGGA	TTGCTCCAGCAAAACCTCCT
unique down_GFP+R	*psmb4*	CGAAAGCCATCCACTCCTGG	CTCATCAGAGGCTGTGCCAA
unique down_GFP+R	*cebpa*	AGTACAGGCTGAGGAGGGAG	CAGTTGCCCATGGCTTTGAC
unique down_GFP+R	*psma5*	TGTGCCATGAGTGGCTTGAT	CGCGTCACACTGGACAAAAG
Reference 1	*mat1a*	GTCTACCATCTGCAGCCCAG	GGCGTAAGACACCTGAACCA
Reference 2	*cox7b*	TATTAGCACGCGCAGTCAGT	GACAGGTTCCATGCAATGCC

Disease Models & Mechanisms • Supplementary information

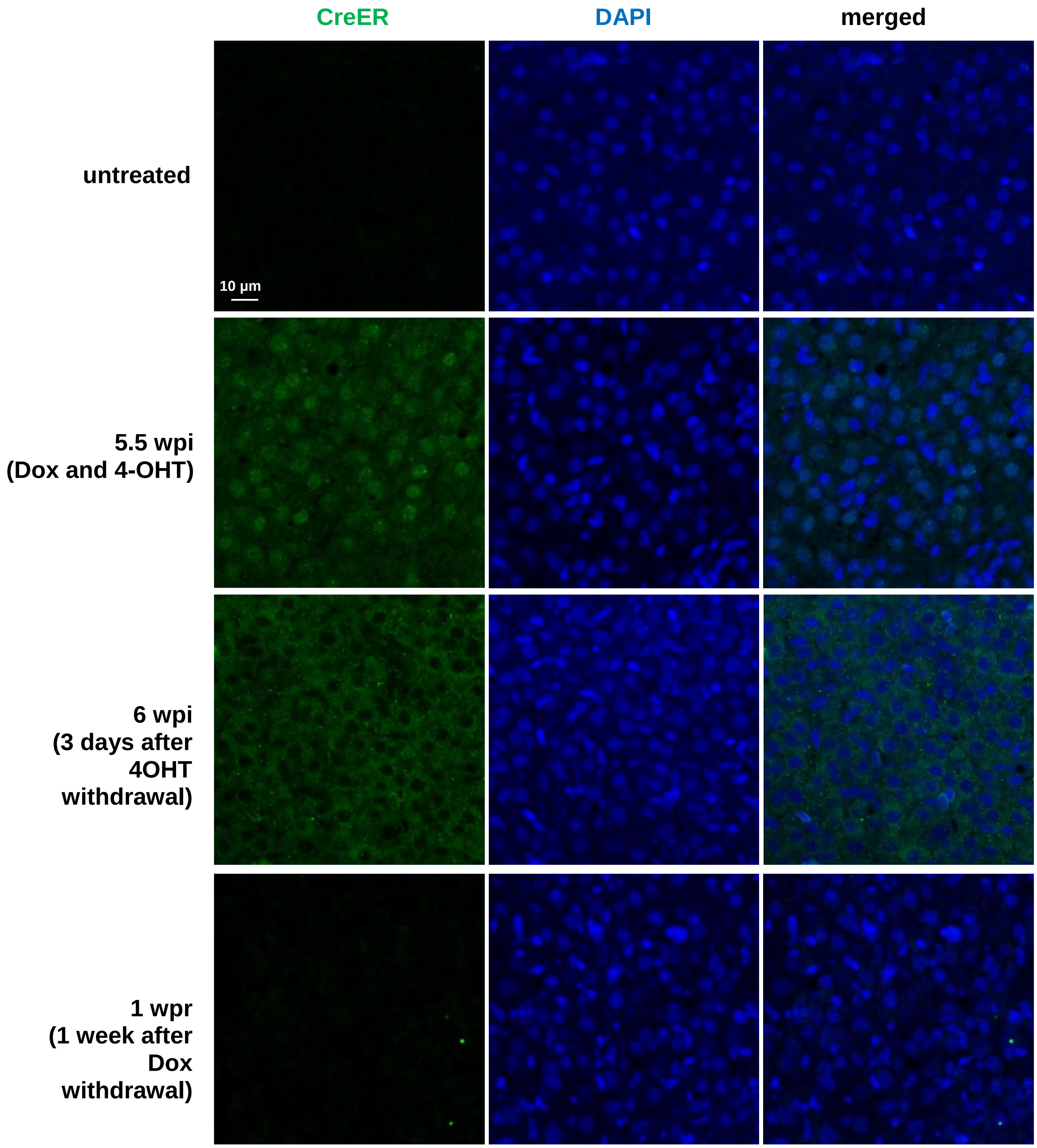

Fig. S1. Immunofluorescent staining of liver sections to validate the expression, cellular localization and stability of CreER. In untreated *CreER/xmrk* fish, no expression of CreER was detected. After Dox and 4-OHT induction at 5.5 wpi, CreER was expressed and was localized into nucleus. After 4-OHT withdrawal at 6 wpi, CreER was localized in the cytoplasm while not in the nucleus anymore. After Dox withdrawal at 1 wpr, CreER was basically undetectable.

Disease Models & Mechanisms • Supplementary information

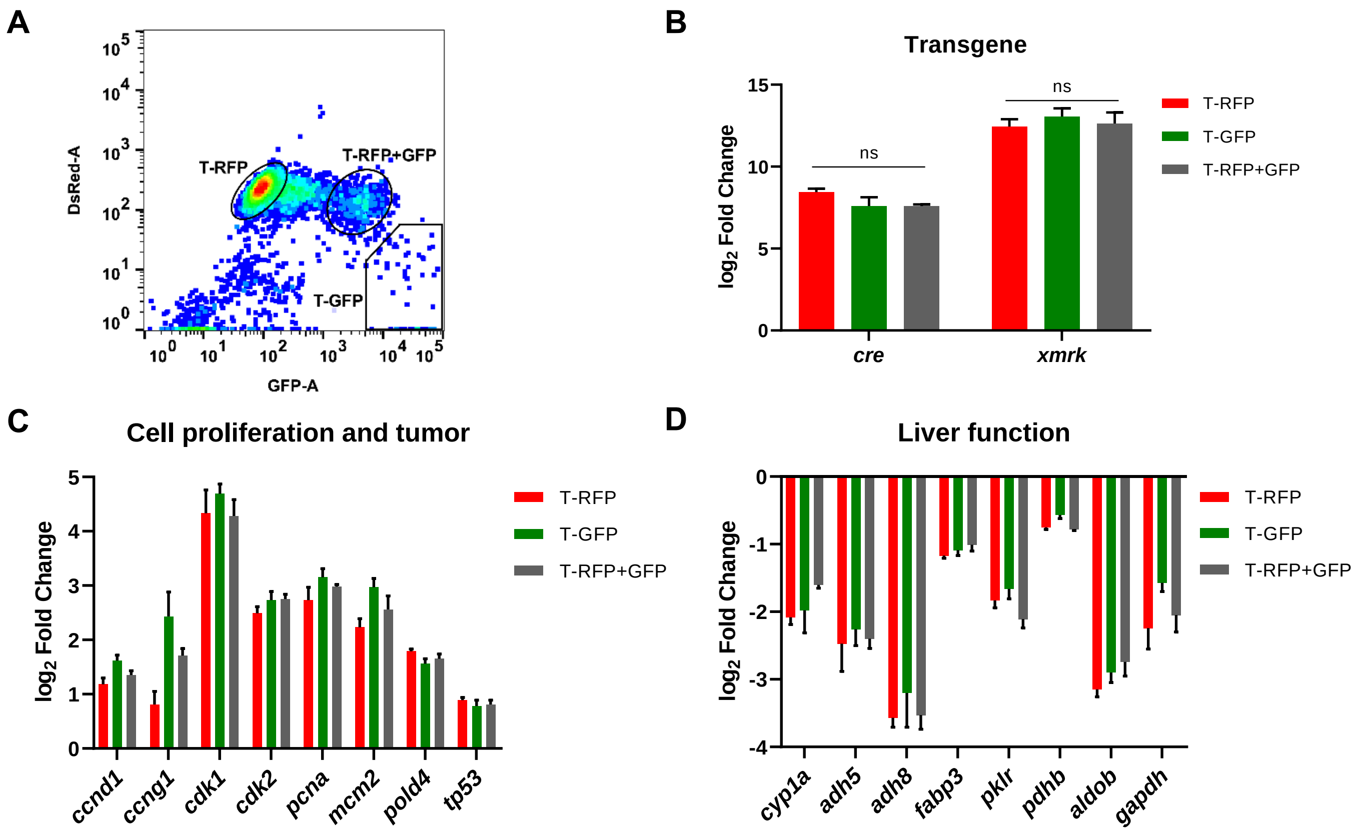

Fig. S2. Comparison of RFP+ (T-RFP), GFP+ (T-GFP), and double-positive (T-RFP+GFP) hepatocytes after Dox and 4-OHT treatments at 6 wpi from *CreER/xmrk* fish. (A) T-GFP cells (which failed to undergo loxP recombination and color switch) and T-RFP+GFP cells (which have undergone loxP recombination but with GFP protein not fully degraded) express comparable levels of *cre* and *xmrk* compared to T-RFP+ cells. (B) Genes involved in cell proliferation and tumor were up-regulated in all three population of cells at comparable levels. (C) Genes that are associated with liver function (detoxification metabolism, lipid and glucose metabolism) were down-regulated in all three population of cells at comparable levels. The fold changes are relative to the levels in the normal control hepatocytes. These results indicate that the T-GFP+ and T-RFP+GFP cells at 6 wpi are molecularly equivalent to T-RFP+ tumor cells. The GFP+ cells are likely the tumor hepatocytes that didn't undergo loxP recombination and color switch, rather than newly differentiated hepatocytes.

Disease Models & Mechanisms • Supplementary information

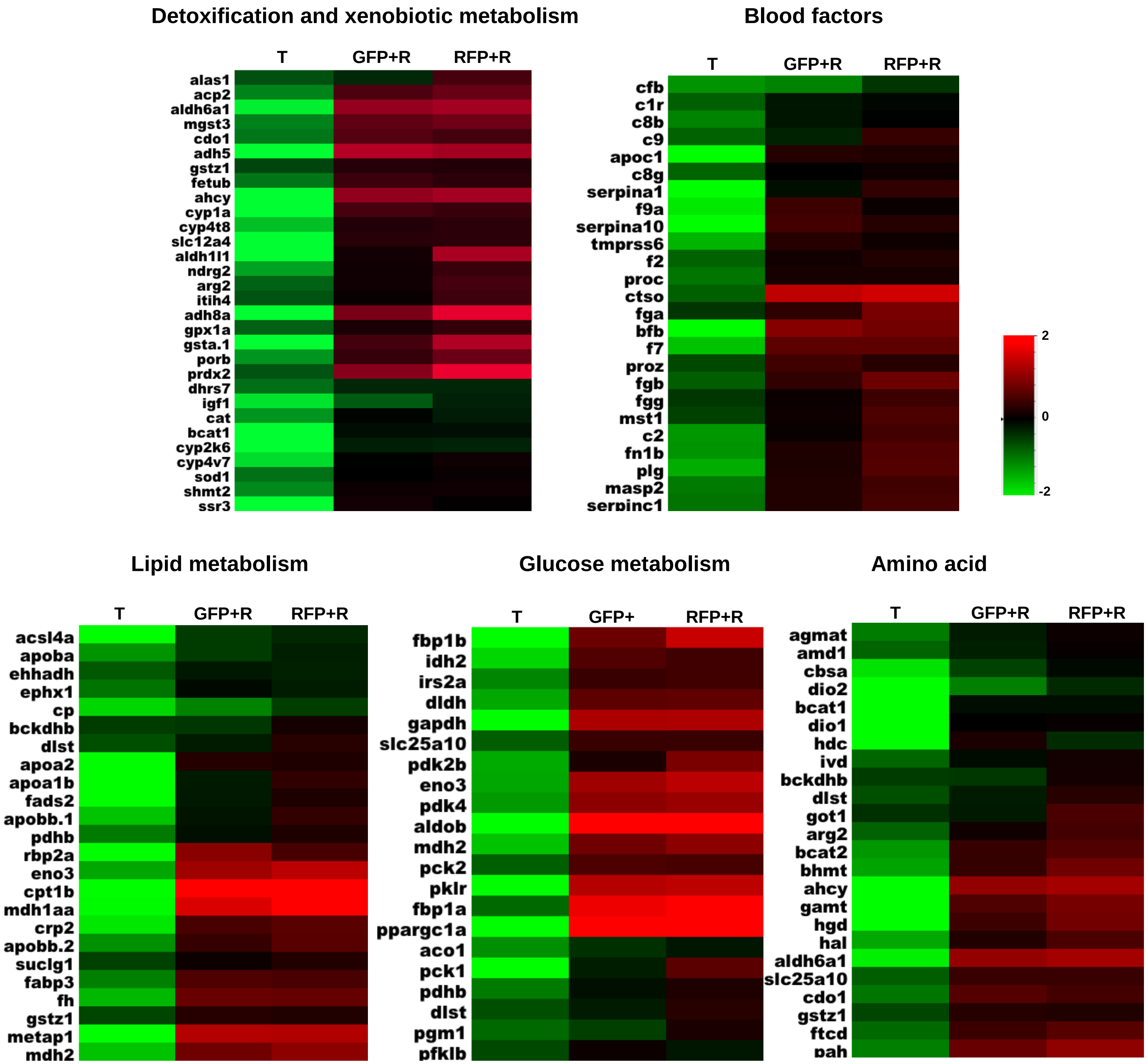

Fig. S3. Expression profiles of selected genes that are associated with liver function: Detoxification and xenobiotic metabolism, Blood factors, Lipid metabolism, Glucose metabolism, and Amino acid metabolism. The expression data was based on RNA-Seq analyses and fold changes are relative to the levels in the normal control samples, i.e. T vs C; GFP+R and RFP+R vs CR. The scale represents log2 Fold Change of 2 to -2.

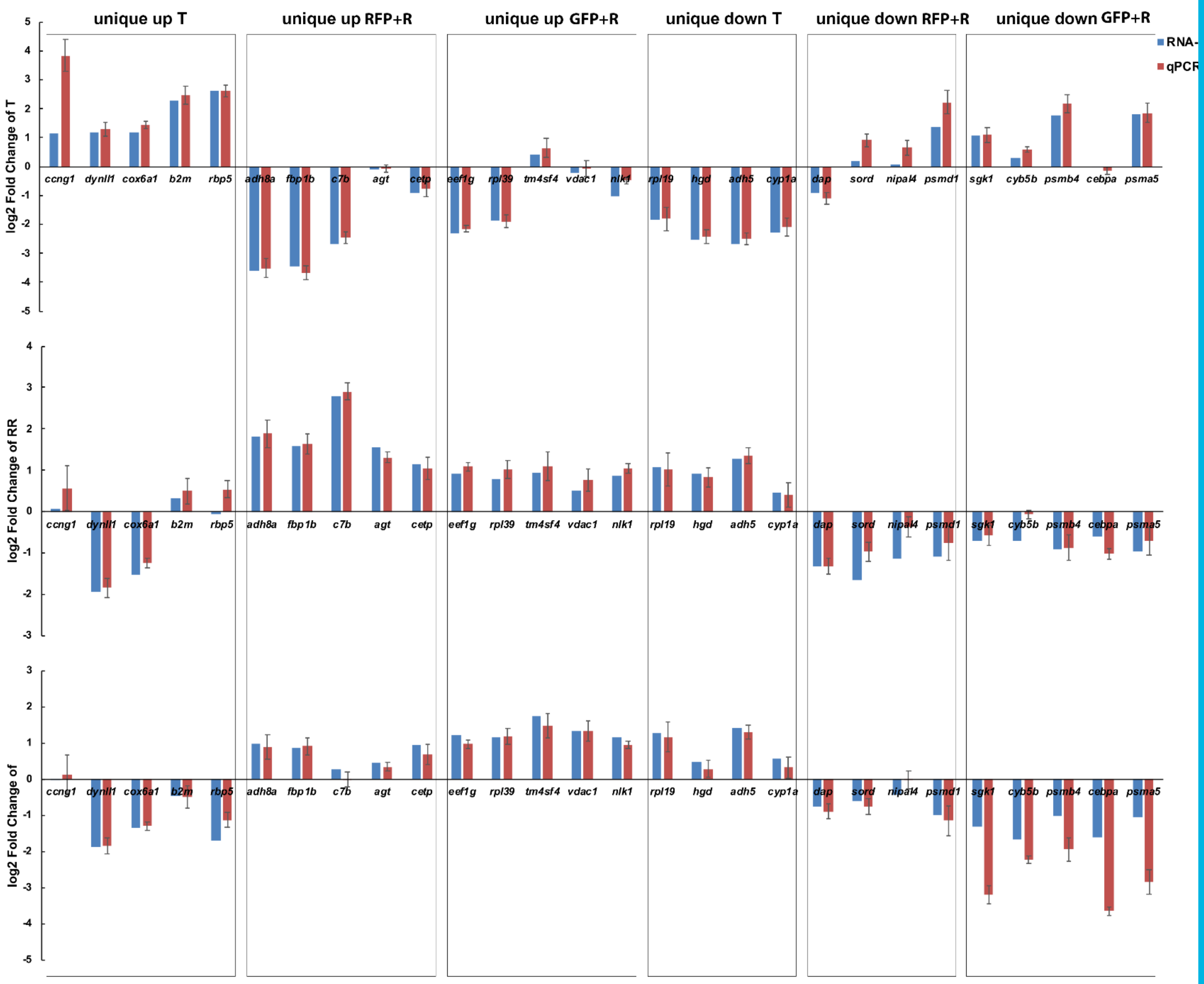

Fig. S4. qPCR validation of RNA-seq data. 4-5 genes were selected from each uniquely upregulated or downregulated category in T, RFP+R and GFP+R, as shown in the Venn diagram of Fig. 5 A-B. Genes with different abundance levels (FPKM 10 to 1000) were covered and majority of the genes tested showed good concordance between RT-qPCR and RNA-seq.
